# Briareolate Esters from the Gorgonian *Briareum asbestinum*

**DOI:** 10.3390/md10081662

**Published:** 2012-08-10

**Authors:** Rian J. Meginley, Prasoon Gupta, Thomas C. Schulz, Amanda B. McLean, Allan J. Robins, Lyndon M. West

**Affiliations:** 1 Department of Chemistry and Biochemistry, Florida Atlantic University, Boca Raton, FL 33431, USA; Email: rmeginle@fau.edu (R.J.M.); pgupta2@fau.edu (P.G.); 2 Viacyte Inc., 111 Riverbend Rd, Athens, GA 30602, USA; Email: TSchulz@viacyte.com (T.C.S.); ARobins@viacyte.com (A.J.R.); 3 Department of Biochemistry and Molecular Biology, The University of Georgia, Athens, GA 30602, USA; Email: abeggs@uga.edu

**Keywords:** *Briareum asbestinum*, octocoral, briarane, briareolate ester, human embryonic stem cell

## Abstract

Two new briarane diterpenoids briareolate esters J (**1**) and K (**2**) were isolated from the methanolic extract of the octocoral *Briareum asbestinum* collected off the coast of Boca Raton, Florida. The structures of briaranes **1** and **2** were elucidated by interpretation of spectroscopic data. Briareolate ester K (**2**) showed weak growth inhibition activity against human embryonic stem cells (BG02).

## 1. Introduction

Gorgonian corals have provided an abundance of novel structures with many of these demonstrating potentially useful biological activities. The gorgonian *Briareum asbestinum* has shown to be a plentiful source of diterpenoids belonging to the eunicellin, asbestinane, cembrane, and briarane classes and display numerous biological activities (e.g., cytotoxicity, antimicrobial, anti-inflammatory, antiviral, immunomodulatory, antifouling, and ichthotoxicity) [1]. Briarane-type diterpenoids contain a highly oxidized bicyclo[8.4.0] system of which most contain a γ-lactone ring. The briareolate esters are a small group of briarane diterpenoids isolated from *Briareum asbestinum* that contain a C-19 methyl ester instead of the typical γ-lactone ring [2,3,4]. 

**Figure 1:**
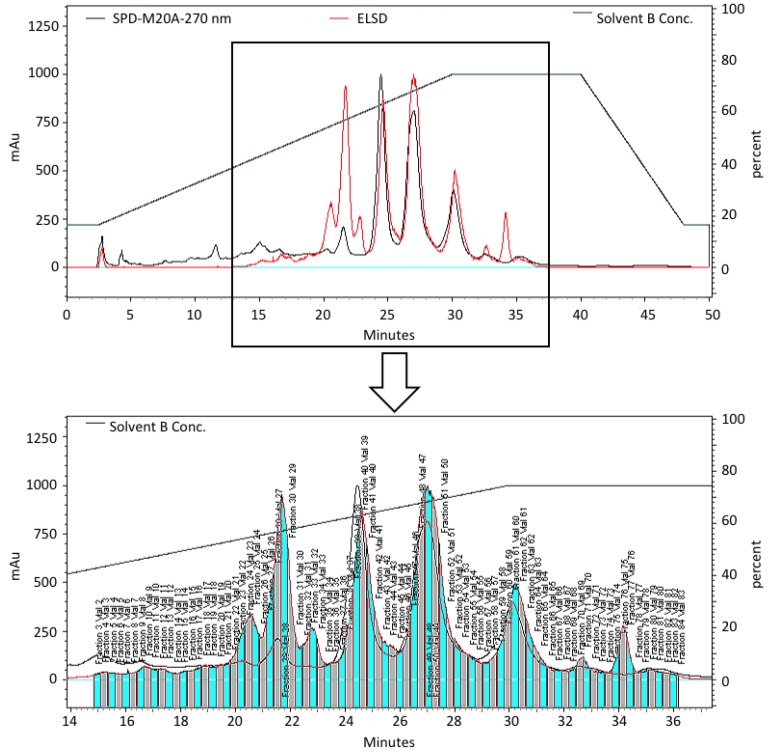
HPLC chromatogram of the pre-fractionated extract of *B. asbestinum* showing the fractions separated using ELSD-directed collection to generate a semi-purified extract library for biological screening.

**Figure 2:**
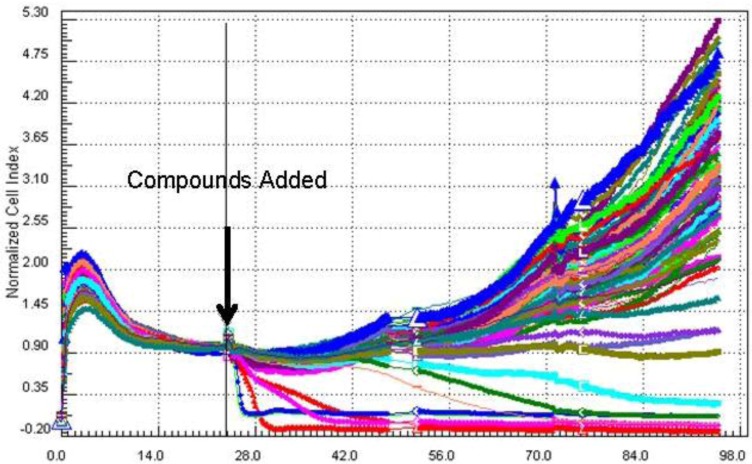
A cell index *vs.* time plot for a pre-fractionated extract library of *B. asbestinum* against the BG02 cell line. A drop in cell index within the first 24–48 h after addition of the compounds is interpreted as toxicity or induction of apoptosis.

As part of an ongoing study to discover compounds that impact human embryonic stem cell (hESC) growth we have been screening pre-fractionated and semi-purified marine natural product extract libraries generated using a solid phase extraction (SPE) procedure followed by semi-preparative high pressure liquid chromatography (HPLC) using evaporative light scattering detection (ELSD) directed fractionation (Figure 1). The cell growth inhibitory activities of the pre-fractionated extract libraries are then evaluated against human embryonic stem cells (BG02) using a 96-well plate real-time cell electronic sensing (RT-CES) system to identify compounds that impact self-renewal, differentiation or apoptosis. A plot of cell index (impedance) *versus* time is used to indicate relative proliferation, differentiation, or death in real-time (Figure 2). 

**Figure 3:**
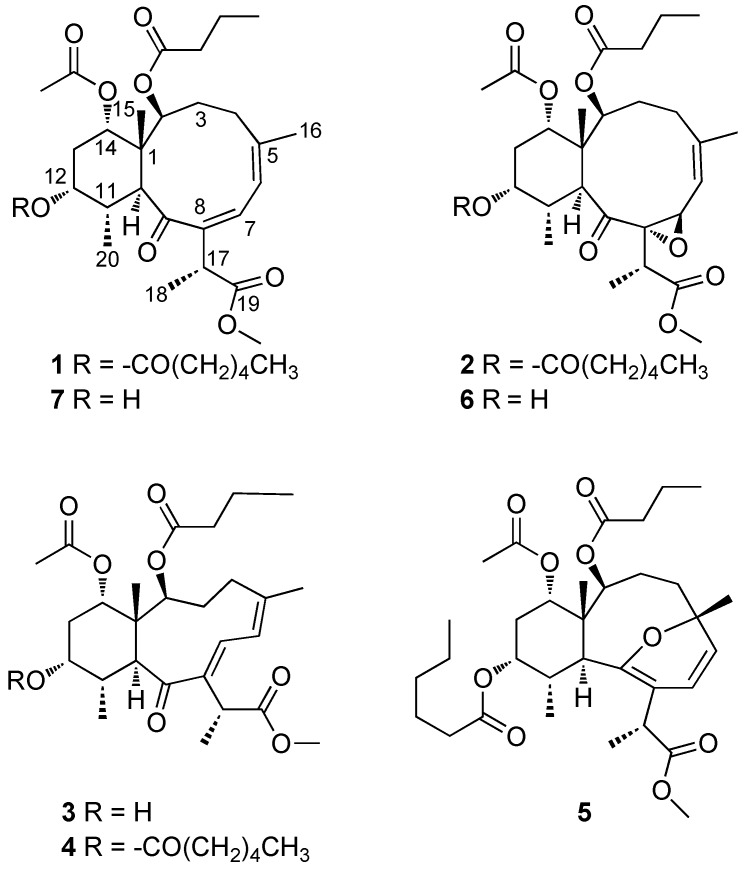
Structures of compounds **1**–**7**.

Our previous work using this approach on *B. asbestinum* resulted in the isolation of three new briareolate esters L–N (**3**–**5**) [5]. This included the biologically active compound briareolate ester L (**3**) that was found to possess a 10-membered macrocyclic ring with a (*E*,*Z*)-dieneone and was shown to contain a “spring loaded” Michael acceptor that is capable of forming a reversible covalent bond to model sulfur-based nucleophiles. In the present study we report the isolation and structural elucidation of two additional new briarane diterpenoids briareolate esters J (**1**) and K (**2**), along with three known compounds from the methanolic extract of *B. asbestinum* that was found to exhibit growth inhibition against BG02 cells (Figure 3).

## 2. Results and Discussion

**Figure 4:**
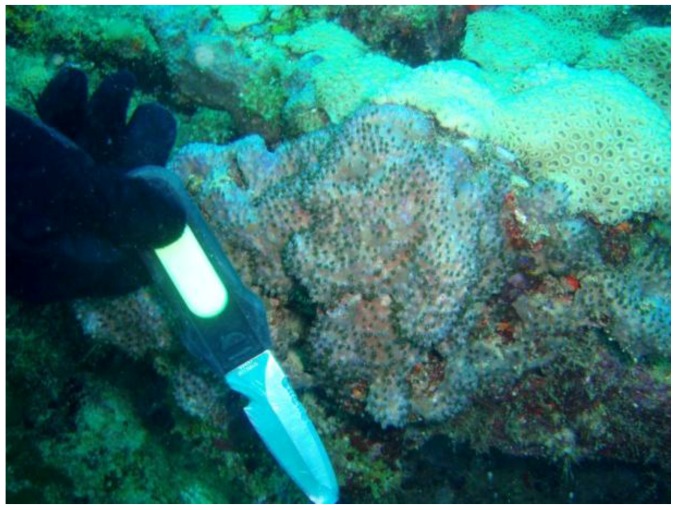
*Briareum asbestinum*.

Specimens of *Briareum asbestinum* (Figure 4) were collected at Hillsboro Ledge, Boca Raton Florida and kept frozen until extraction. The methanolic extract was first fractionated on polymeric HP-20 resin using cyclic loading [6]. The HP-20 column was eluted with 800 mL fractions of (1) H_2_O, (2) 40% Me_2_CO/H_2_O, (3) 75% Me_2_CO/H_2_O and (4) Me_2_CO. The Me_2_CO fraction was then subjected to column chromatography on HP-20SS and normal phase HPLC to yield two new briareolate esters J (**1**) and K (**2**) and three known briareolate esters D (**6**), G (**7**), and M (**4**).

**Figure 5:**
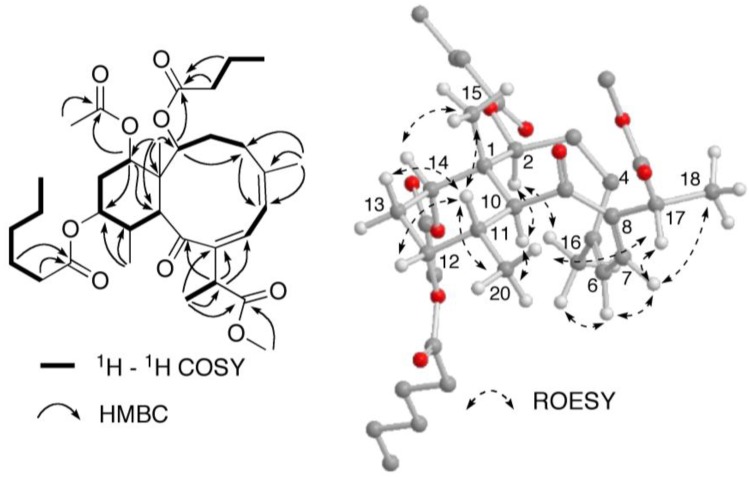
Selected 2D NMR correlations for briareolate ester J (**1**).

Briareolate ester J (**1**) was isolated as a colorless oil. The molecular formula of briareolate ester J (**1**), C_33_H_50_O_9_, was determined from the HRESIMS of the [M + Na]^+^ ion at *m*/*z* 613.3346, 98 mass units higher than that of briareolate ester G (**7**). The presence of a ketone conjugated with two double bonds (α,β,γ,δ-unsaturated ketone) was indicated from a carbonyl carbon with a chemical shift of δ_C_ 207 (C-9), and the C-C double bond carbons [δ_C_ 146.3 (C-5), δ_C_ 125.3 (C-6), δ_C_ 140.9 (C-7), δ_C_ 146.3 (C-8)]. The observation of absorption maxima at λ_max_ = 287 and 225 nm in the UV spectrum was consistent with this assignment. NOE correlations observed from the olefinic proton H-6 to H-7 and H_3_-16, together with correlations from H-7 to both H-17 and H_3_-18, established the (*Z*,*Z*)-configuration of the dieneone and the *s*-*cis* conformation of the diene (Figure 5). Additionally, the upfield chemical shift of the olefinic proton H-7 in **1 **at δ_H_ 6.74 compared toδ_H_ 7.64 in the (*E*,*Z*)-dieneone briareolate ester L (**3**), with the *s*-*trans* diene conformation, confirmed this assignment [5]. 

A close inspection of the ^1^H and ^13^C NMR data (Table 1) revealed the similarity of **1** to that of briareolate ester G (**7**), except that H-12 [δ_H_ 4.89, br s] was shifted downfield by 1.24 ppm as compared with that of **1**. In addition, in the ^13^C NMR spectrum the resonance of C-12 (δ_C_ 73.7) was shifted downfield by 2.7 ppm and those of C-11 (δ_C_ 38.4) and C-13 (δ_C_ 30.2) were shifted upfield by 0.5 and 1.3 ppm, respectively, in comparison with those of **1** [4]. This suggested that the 12-hydroxy group of **7**, was replaced by a hexanoate group at C-12 in **1**, as observed in briareolate esters M (**4**) and N (**5**). The presence of the hexanoate group was confirmed by the NMR data [δ_H_ 0.92 (3H, t, *J* = 8.0 Hz), *ca.* 1.31 (4H, overlapped), 1.63 (2H, overlapped), 2.32 (2H, m), δ_C_ 17.6 (q), 24.0, 26.3, 33.0, and 36.1 (each t), 175.1 (CO)]. These assignments were confirmed by COSY, HSQC, and HMBC correlations similar to those observed for **4**, **5** and **7** (Figure 5). The relative configuration of briareolate ester J (**1**) was determined to be the same as that of **7** from the similarity of proton-proton coupling constants and ^1^H and ^13^C chemical shifts together with NOE correlations observed in a ROESY experiment (Figure 5). 

**Table 1:** NMR Spectroscopic Data for Briareolate Esters J (**1**) and K (**2**) ^a^.

position	1		2	
	δ_C_, mult	δ_H_ ( *J* in Hz)	δ_C_, mult	δ_H_ ( *J* in Hz)
1	46.4, C		48.8, C	
2	73.7, CH	5.17, br d (6.0)	80.5, CH	5.52, d (8.0)
3α	31.3, CH_2_	1.89, m	31.1, CH_2_	2.31, m
3β		1.89, m		1.58, m
4α	30.2, CH_2_	2.20, m	34.1, CH_2_	2.53, dd (18.0, 8.0)
4β		2.08, m		2.28, m
5	146.3, C		147.1, C	
6	125.3, CH	6.12, br s	116.2, CH	5.47, br d (4.0)
7	140.9, CH	6.74, br s	64.3, CH	4.42, br d (4.0)
8	146.3, C		71.3, C	
9	207.0, C		211.5, C	
10	48.3, CH	3.79, d (12.0)	44.7, CH	3.06, d (12.0)
11	38.4, CH	2.19, m	36.2, CH	2.34, m
12	73.7, CH	4.89, br s	72.6, CH	4.98, br s
13α	30.2,CH_2_	2.05, m	29.9, CH_2_	2.05, br d (16.0)
13β		1.86, m		1.99, m
14	75.6, CH	4.68, br s	74.6, CH	4.69, br s
15	14.9, CH_3_	1.19, s	12.8, CH_3_	0.99, s
16	26.3, CH_3_	2.19, s	26.8, CH_3_	1.75, s
17	46.4, CH	3.45, q (8.0)	40.8, CH	2.39, q (8.0)
18	19.8, CH_3_	1.32, d (8.0)	13.5, CH_3_	1.29, d (8.0)
19	176.7, C		175.3, C	
20	17.6, CH_3_	0.79, d (8.0)	16.8, CH_3_	0.75, d (8.0)
OMe	52.8, CH_3_	3.62, s	52.5, CH_3_	3.61, s
ester at C-2	175.1, C		174.3, C	
	37.7, CH_2_	2.19, m	37.9, CH_2_	2.25, m
	19.2, CH_2_	1.57, m	19.7, CH_2_	1.60, m
	14.5, CH_3_	0.92, t (8.0)	14.5, CH_3_	0.93, t (8.0)
ester at C-12	175.1, C		175.4, C	
	36.1, CH_2_	2.32, m	35.9 CH_2_	2.37, m
	26.3, CH_2_	1.63, m	26.4 CH_2_	1.64, m
	33.0, CH_2_	1.31, m	33.0 CH_2_	1.33, m
	24.0, CH_2_	1.31, m	24.0 CH_2_	1.33, m
	17.6, CH_3_	0.92, t (8.0)	14.8 CH_3_	0.95, t (8.0)
ester at C-14	172.1, C		172.6, C	
	22.1, CH_3_	1.98, s	22.3 CH_3_	1.99, s

^a^ In CD_3_OD, 400 MHz for ^1^H and 100 MHz for ^13^C NMR.

**Figure 6:**
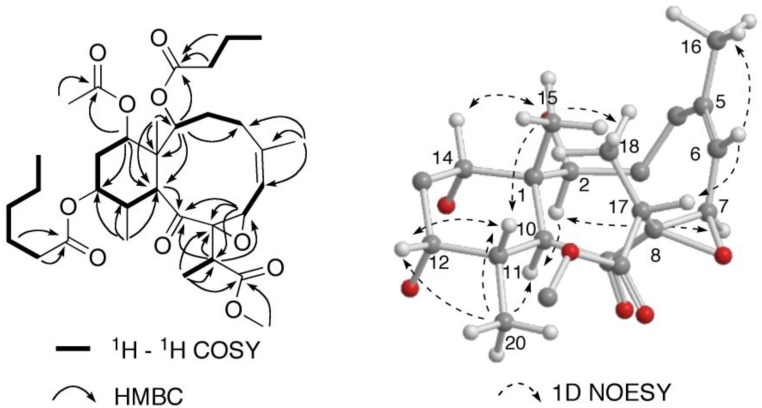
Selected NMR correlations for briareolate ester K (**2**).

Briareolate ester K (**2**) was isolated as a colorless oil. The molecular formula of briareolate ester K (**2**), C_33_H_50_O_10_, was determined from the HRESIMS of the [M + Na]^+^ ion at *m*/*z* 629.3291, one more oxygen atom than that of briareolate ester J (**1**). A comparison of the ^1^H and ^13^C NMR data (Table 1) revealed that **2** was similar to **1**, except that the NMR signals for the C-7–C-8 double bond were missing in the NMR spectra of **2**, and instead, resonances for an epoxide were observed in the ^1^H NMR (δ 4.42, H-6) and ^13^C NMR [δ_C_ 64.3 (s); 71.3 (d)] spectra indicating oxidation of the C-7–C-8 double bond to be an epoxide. HMBC correlations from the oxygenated methine signal at δ 4.42 (H-7) to C-5 (δ 147.1) and C-6 (δ 116.2) of the trisubstituted double bond, and to C-8 (δ 71.3) and C-9 (δ 211.5) are consistent with this assignment. Additional HMBC correlations observed from H-17 to C-7, C-8 and C-9 confirmed this assignment (Figure 6). 

The relative configuration of briareolate ester K (**2**) was determined to be identical to that of **1** on the basis of NOE enhancements revealed in a series of 1D NOESY experiments (Figure 6). The configuration of the C-7/8 epoxide was determined from NOE enhancements observed between H-7 and H-2 together with NOE enhancements observed from H-6 to H-17 which placed H-7 on the inside of the 10-membered ring and indicated the *trans* orientation of the epoxide. This assignment is consistent with that of briareolate ester D (**6**) whose structure was assigned on the basis of X-ray crystallography [4]. 

Compounds **1** and **2** were evaluated for cell growth inhibitory activities against human embryonic stem cells (BG02) using a 96-well plate real-time cell electronic sensing (RT-CES) system [7]. Briareolate ester K (**2**) showed weak growth inhibition against BG02 cells with an EC_50_ value of 40 µM. No inhibitory activity was detected for briareolate ester J (**1**) at 40 µM. Previously the (*E*,*Z*)-dienone containing briareolate esters L (**3**) and M (**4**) were found to have growth inhibition against both the BG02 and a pancreatic cancer cell line (BxPC-3) cells with EC_50_ values of 2.4 and 9.3 µM, respectively for **3**, and 8.0 µM against BG02 cells and only cytostatic effects at 13.0 and 17.0 µM against the BxPC-3 cells for **4 **[5]. No growth inhibition was found for the briareolate esters B, C, G (**7**), and N (**5**). The absence of significant growth inhibition activity for the (*Z*,*Z*)-dieneone compound **1** and the epoxide containing compound **2** further confirms the importance of the (*E*,*Z*)-dieneone for biological activity.

## 3. Experimental Section

### 3.1. General Experimental Procedures

Optical rotations were measured on a Jasco P-2000 polarimeter (*c*: g/100 mL) at 589 nm. UV spectra were obtained on a Perkin-Elmer Lambda EZ 210 UV-vis spectrophotometer. IR spectra were recorded on a Thermo Electronic Corporation Nicolet IR-100 spectrophotometer. All NMR spectra were recorded on a Varian MercuryPlus 400 spectrometer. All chemical shifts (δ) were referenced internally to the residual solvent peak (CD_3_OD: ^1^H, δ 3.30; ^13^C, δ 49.0; CDCl_3_: ^1^H 7.26 ppm; ^13^C 77.0 ppm). Short- and long-range ^1^H–^13^C correlations were determined with gradient-enhanced inverse-detected HSQC and HMBC experiments respectively. NOE correlations were detected with NOESY or ROESY experiments with a 0.5 s mixing time. The high-resolution ESI mass spectra were obtained using an Agilent 6210 LC-TOF mass spectrometer at the Mass Spectrometer Facility at the University of Florida, Gainesville, Florida. HPLC purifications were performed on Beckman System Gold HPLC system with a 168 UV detector and a SEDEX 85 (Sedere) evaporative light scattering detector. Thin layer chromatography (TLC) analyses were performed using Merck Kieselgel (Aufoilen) 60 F_254_ plates. TLC plates were visualized by spraying with 1:1 MeOH:H_2_SO_4_.

### 3.2. Animal Material

The gorgonian *Briareum asbesinum* was collected by hand using SCUBA at a depth of 45–50 ft from Hillsboro Ledge, Boca Raton Florida. The specimen was immediately frozen and kept at −20 °C until extraction. A voucher specimen has been deposited in the Department of Chemistry and Biochemistry, Florida Atlantic University, Boca Raton Florida (FAU02-010). 

### 3.3. Extraction and Isolation

The sample of *Briareum asbestinum* (500 g wet wt.), were extracted with MeOH (3 × 800 mL) for 24 h. The third, second and then the first extracts were passed through a column of HP-20 resin (2.5 × 25 cm) equilibrated with MeOH. The combined eluents was diluted with H_2_O (2.5 L) and passed again through the column. The column was eluted with 800 mL fractions of: (1) H_2_O, (2) 40% Me_2_CO/H_2_O, (3) 75% Me_2_CO/H_2_O and (4) Me_2_CO. Fraction 4 was concentrated to dryness and was subjected to column chromatography on HP-20SS resin eluting with increasing concentrations of MeCN in H_2_O (40%–100%). A late eluting fraction was further subjected to semi-preparative silica gel HPLC (Luna 5 µm; 10 × 250 mm; 4 mL/min; 5%–50% EtOAc/Hexane over 30 min) to give **1** (6.0 mg), **2** (10.0 mg), **4** (10.0 mg), **6** (10.0 mg) and **7** (5.0 mg). 

Briareolate ester J (**1**): Colorless oil; [α]^25^_D_ −19 (*c* 0.05, MeOH); UV (MeOH) λ_max_ 225 nm (ε 2711), 287 (ε 1713); IR (MeOH) ν_max_ 2956, 2867, 1737, 1641, 1454, 1371 cm^−1^; ^1^H and ^13^C NMR data, see Table 1; HRESIMS *m/z* 613.3346 [M + Na]^+^ (calcd. for C_33_H_50_O_9_Na, 613.3353).

Briareolate ester K (**2**): Colorless oil; [α]^25^_D_ −8 (*c* 0.05, MeOH); IR (MeOH) ν_max_ 2951, 2869, 1742, 1454, 1367 cm^−1^; ^1^H and ^13^C NMR data, see Table 1; HRESIMS *m/z* 629.3291 [M + Na]^+^ (calcd. for C_33_H_50_O_10_Na, 629.3302).

### 3.4. Cell Culture

BG02 hESCs were grown in defined medium, containing 10 ng/mL HRG1β (Peprotech), 10 ng/mL ActA (R&D Systems), 200 ng/mL LR^3^-IGF1 (JRH Biosciences), and 8 ng/mL FGF2 (Sigma or R&D Systems). Cultures were passaged with Accutase (Innovative Cell Technologies) and plated on tissue culture flasks coated with growth factor-reduced matrigel (BD Biosciences) diluted 1:200, as described by Robins and Schulz [8].

### 3.5. RT-CES Cytotoxicity Assays

The xCELLigence real-time impedance system (Roche) was used to monitor the effects of compounds on cells [7]. BG02 cells were plated at 1 × 10^4^ cells/well in matrigel coated plates. The culture was placed in the reader station under standard humidified conditions and incubated at 37 °C with 5% CO_2_. The media was changed every 24 h and impedance was measured every 15 min for 3 days. Compounds were added to quadruplicate wells 24 h after plating. Vincristine was used as a positive control for cytotoxicity, and DMSO alone was used as a negative control. Cell index plots were normalized immediately prior to addition of compound, and EC_50_ values were generated using the xCELLigence analysis software RTCA ver 1.1 after 2 days of treatment.

## 4. Conclusions

Two new briarane diterpenoids briareolate esters J (**1**) and K (**2**), along with three known compounds were isolated from the methanolic extract of *B. asbestinum* collected off the coast of Boca Raton, Florida. The compounds were identified from screening semi-purified and pre-fractioned extract libraries against hESCs and briareolate ester K (**2**) containing a *trans* C-7/8 epoxide was found to exhibit weak growth inhibition activity against human embryonic stem cells (BG02). The lack of biological activity for briaranes **1** and **2** further confirmed the requirement of the α,β,γ,δ-unsaturated ketone in conjunction with the double bond configuration for biological activity.

## Supplementary Files

Supplementary File 1: PDF-Document (PDF, 681 KB)

## References

[1] Berrue F., Kerr R.G. (2009). Diterpenes from gorgonian corals. Nat. Prod. Rep..

[2] Maharaj D., Mootoo B.S., Lough A.J., McLean S., Reynolds W.F., Tinto W.F. (1992). Methyl briareolate, the first briarein diterpene containing a C-19 methyl ester. Tetrahedron Lett..

[3] Dookran R., Maharaj D., Mootoo B.S., Ramsewak R., McLean S., Reynolds W.F., Tinto W.F. (1994). Briarane and asbestinane diterpenes from *Briareum asbestinum*. Tetrahedron.

[4] Mootoo B.S., Ramsewak R., Sharma R., Tinto W.F., Lough A.J., McLean S., Reynolds W.F., Yang J.P., Yu M. (1996). Further briareolate esters and briareolides from the Caribbean gorgonian octocoral *Briareum asbestinum*. Tetrahedron.

[5] Gupta P., Sharma U., Schulz T.C., Sherrer E.S., McLean A.B., Robins A.J., West L.M. (2011). Bioactive diterpenoid containing a reversible “spring-loaded” (*E,Z*)-dieneone michael acceptor. Org. Lett..

[6] Houssen W.E., Jaspars M., Sarker S.D., Latif Z., Gray A.I. (2006). Methods in Biotechnology. Natural Products Isolation.

[7] Solly K., Wang X., Xu X., Strulovici B., Zheng W. (2004). Application of real-time cell electronic sensing (RT-CES) technology to cell-based assays. Assay Drug Dev. Technol..

[8] Robins A.J., Schulz T.C., Lakshmipathy U., Chesnut J.D., Thyagarajan B. (2009). Media and Extra Cellular Matrix Requirements for Large-Scale ESC Growth. Emerging Technology Platforms for Stem Cells.

