# Supplementary material for: Briareolate Esters from the Gorgonian Briareum asbestinum
Source: Mar Drugs. 2012 Aug 10;10(8):1662–70. doi: 10.3390/md10081662 (PMC3447333; doi:10.3390/md10081662)

# Supplementary Information

**Table of Contents** **Page No**


| Table of Contents | Page No |
|---|---|
| **Figure S1.** $^{1}$H NMR Spectrum of Briareolate Ester J (**1**) ($CD_3OD$, 400 MHz) | 2 |
| **Figure S2.** $^{13}$C NMR Spectrum of Briareolate Ester J (**1**) ($CD_3OD$, 100 MHz) | 3 |
| **Figure S3.** gCOSY NMR Spectrum Briareolate Ester J (**1**) ($CD_3OD$, 400 MHz) | 4 |
| **Figure S4.** gHMBC NMR Spectrum of Briareolate Ester J (**1**) ($CD_3OD$, 400 MHz) | 5 |
| **Figure S5.** ROESY NMR Spectrum of Briareolate Ester J (**1**) ($CD_3OD$, 400 MHz) | 6 |
| **Figure S6.** $^{1}$H NMR Spectrum of Briareolate Ester K (**2**) ($CD_3OD$, 400 MHz) | 7 |
| **Figure S7.** $^{13}$C NMR Spectrum of Briareolate Ester K (**2**) ($CD_3OD$, 100 MHz) | 8 |
| **Figure S8.** gCOSY NMR Spectrum of Briareolate Ester K (**2**) ($CD_3OD$, 400 MHz) | 9 |
| **Figure S9.** gHSQC NMR Spectrum of Briareolate Ester K (**2**) ($CD_3OD$, 400 MHz) | 10 |
| **Figure S10.** gHMBC NMR Spectrum of Briareolate Ester K (**2**) ($CD_3OD$, 400 MHz) | 11 |

**Figure S1.** $^{1}$H NMR Spectrum of Briareolate Ester J (**1**) ($CD_3OD$, 400 MHz).

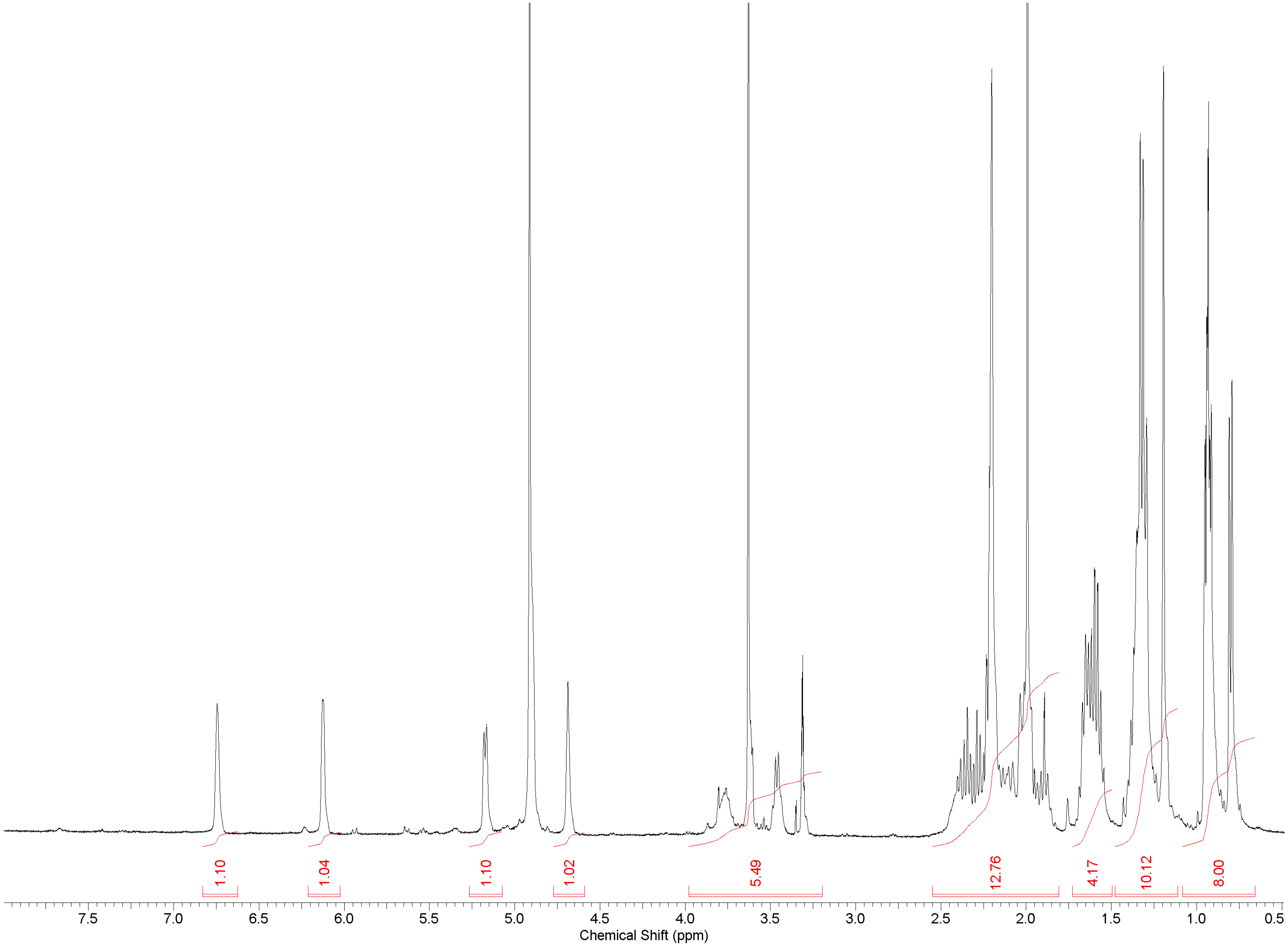

**Figure S2.** $^{13}$C NMR Spectrum of Briareolate Ester J (**1**) ($CD_3OD$, 100 MHz).

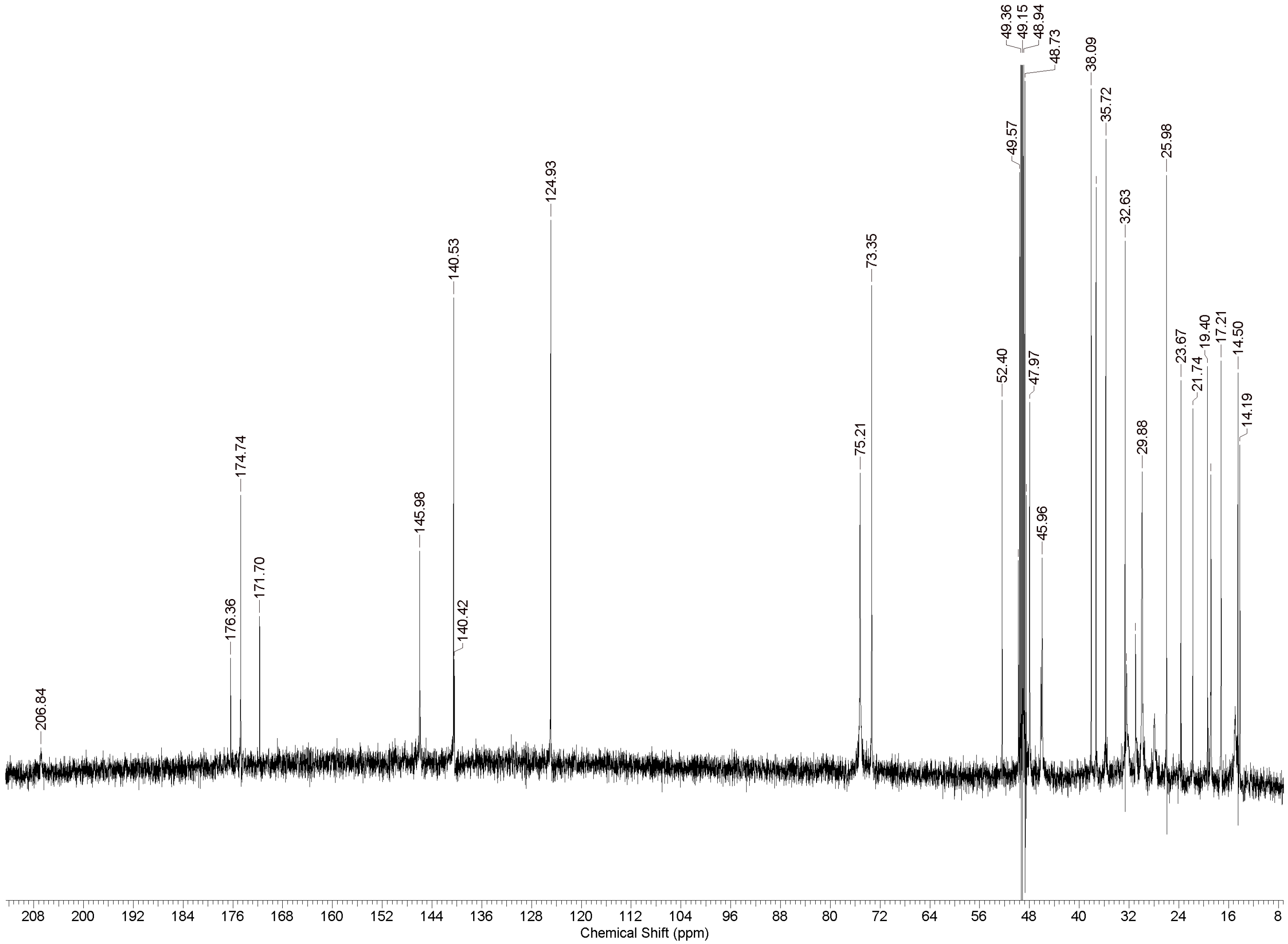

**Figure S3.** gCOSY NMR Spectrum Briareolate Ester J (**1**) ($CD_3OD$, 400 MHz).

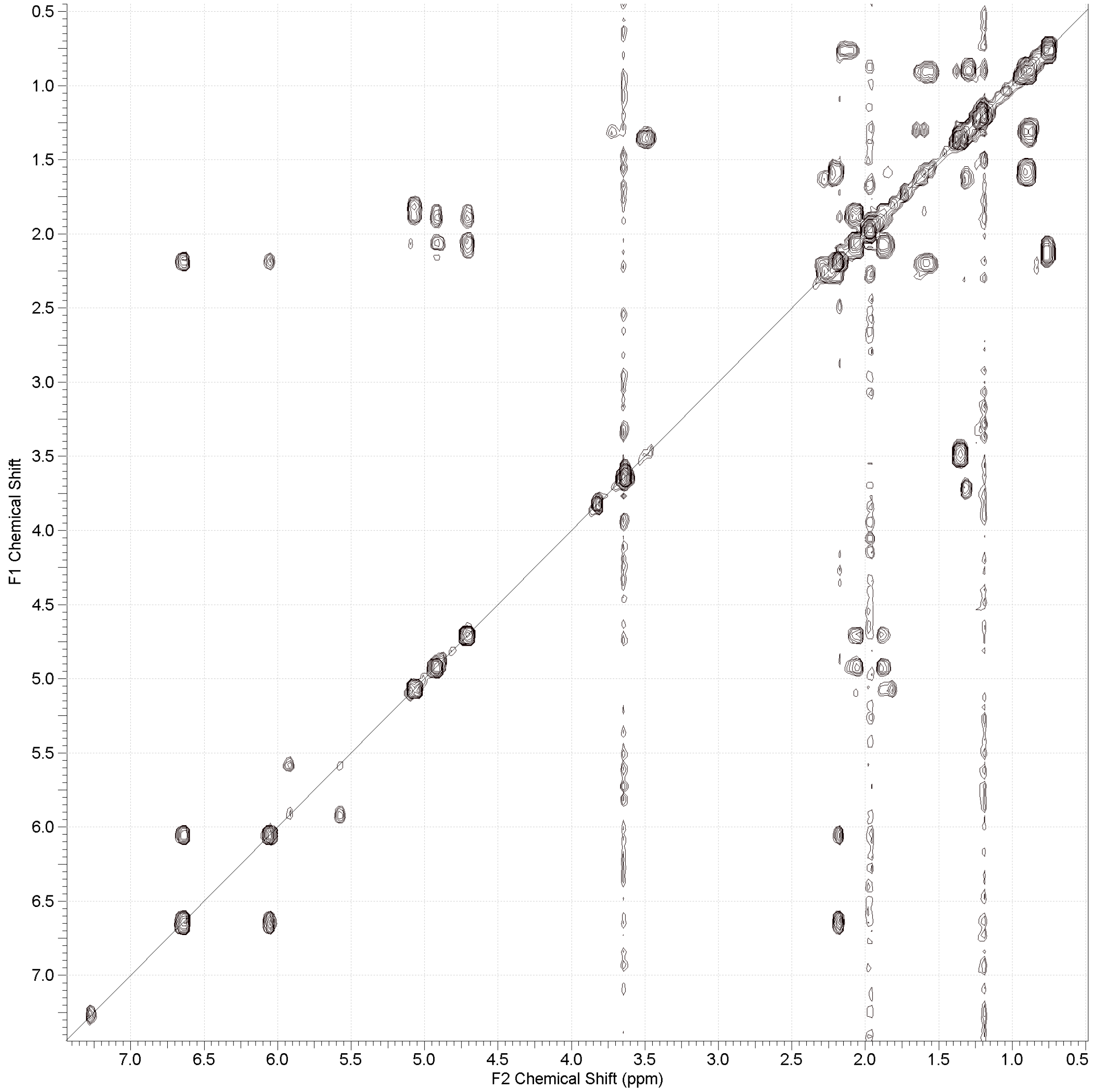

**Figure S4.** gHMBC NMR Spectrum of Briareolate Ester J (**1**) ($CD_3OD$, 400 MHz).

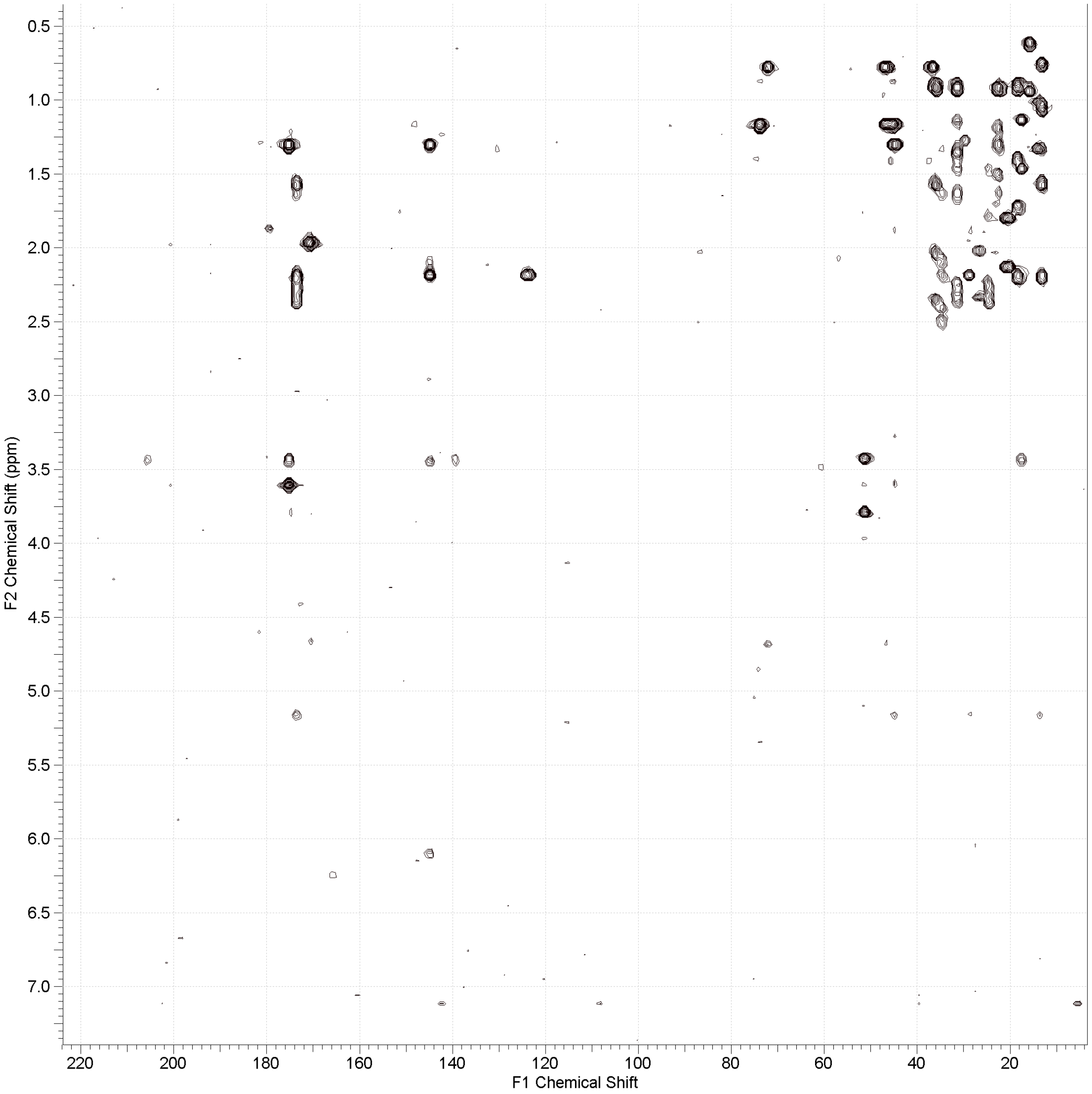

**Figure S5.** ROESY NMR Spectrum of Briareolate Ester J (**1**) ($CD_3OD$, 400 MHz).

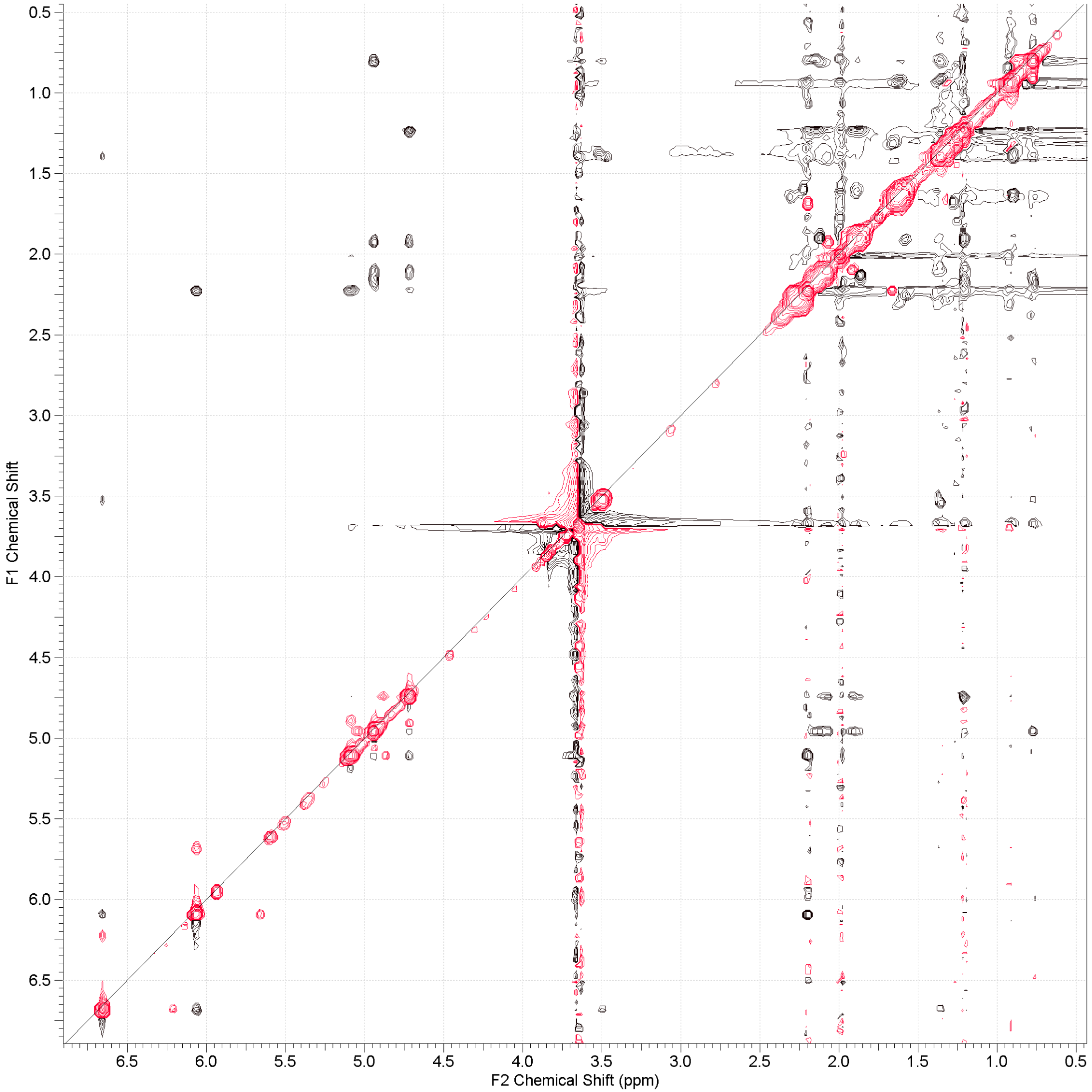

**Figure S6.** $^{1}$H NMR Spectrum of Briareolate Ester K (**2**) ($CD_3OD$, 400 MHz).

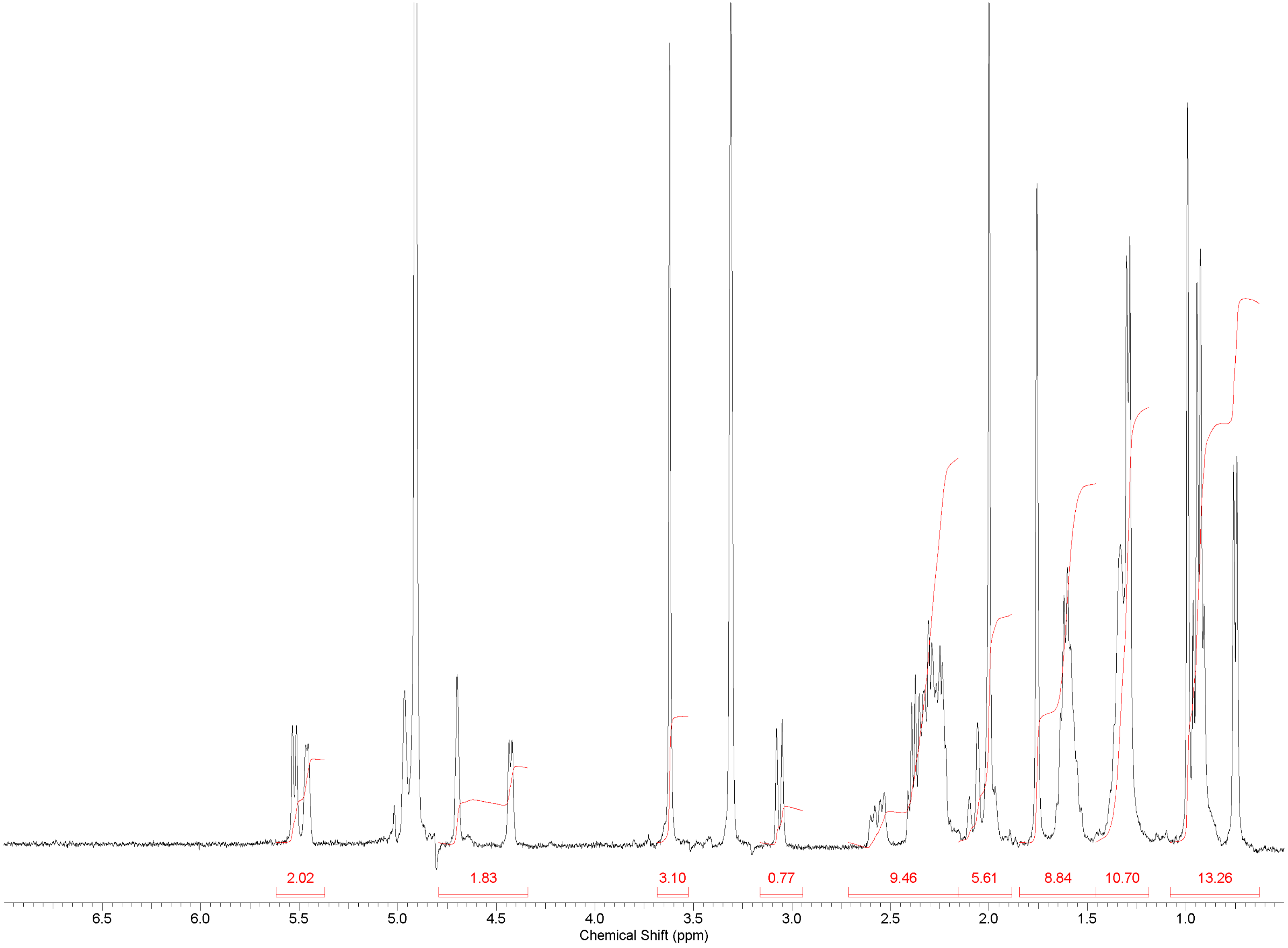

**Figure S7.** gCOSY NMR Spectrum Briareolate Ester K (**2**) ($CD_3OD$, 400 MHz).

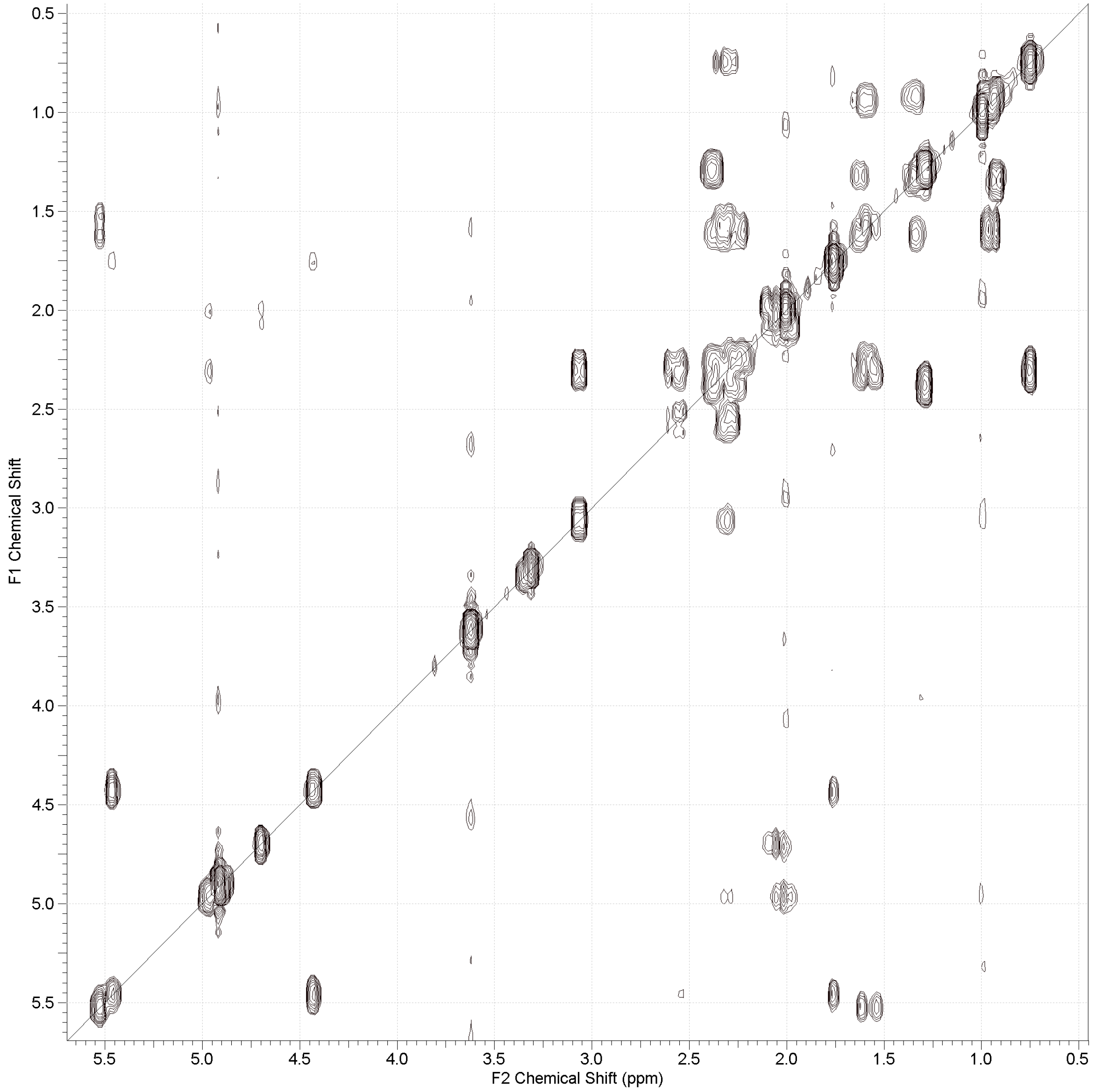

**Figure S8.** $^{13}C$ NMR Spectrum of Briareolate Ester K (**2**) ($CD_3OD$, 100 MHz).

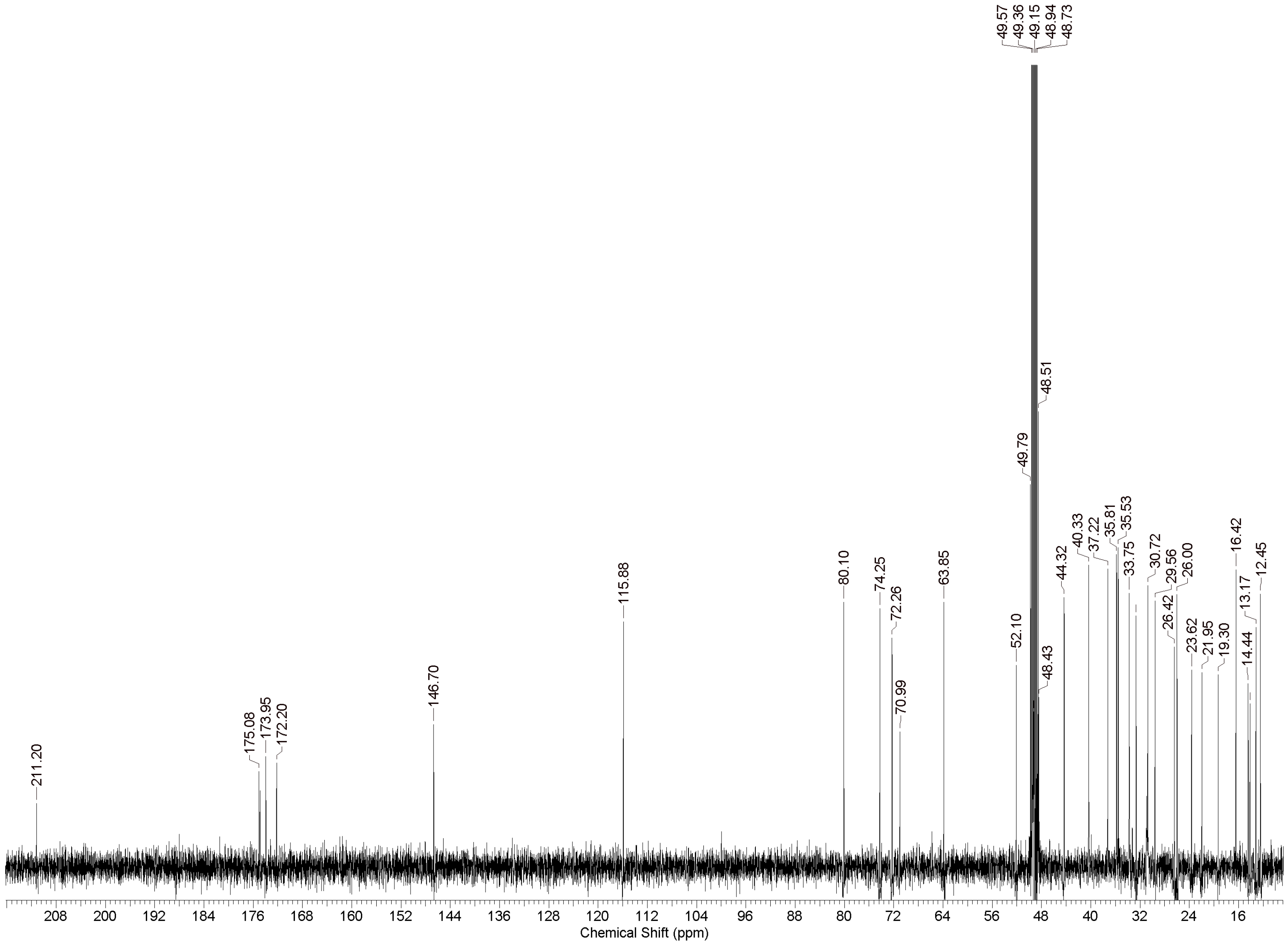

**Figure S9.** gHSQC NMR Spectrum of Briareolate Ester K (**2**) ($CD_3OD$, 400 MHz).

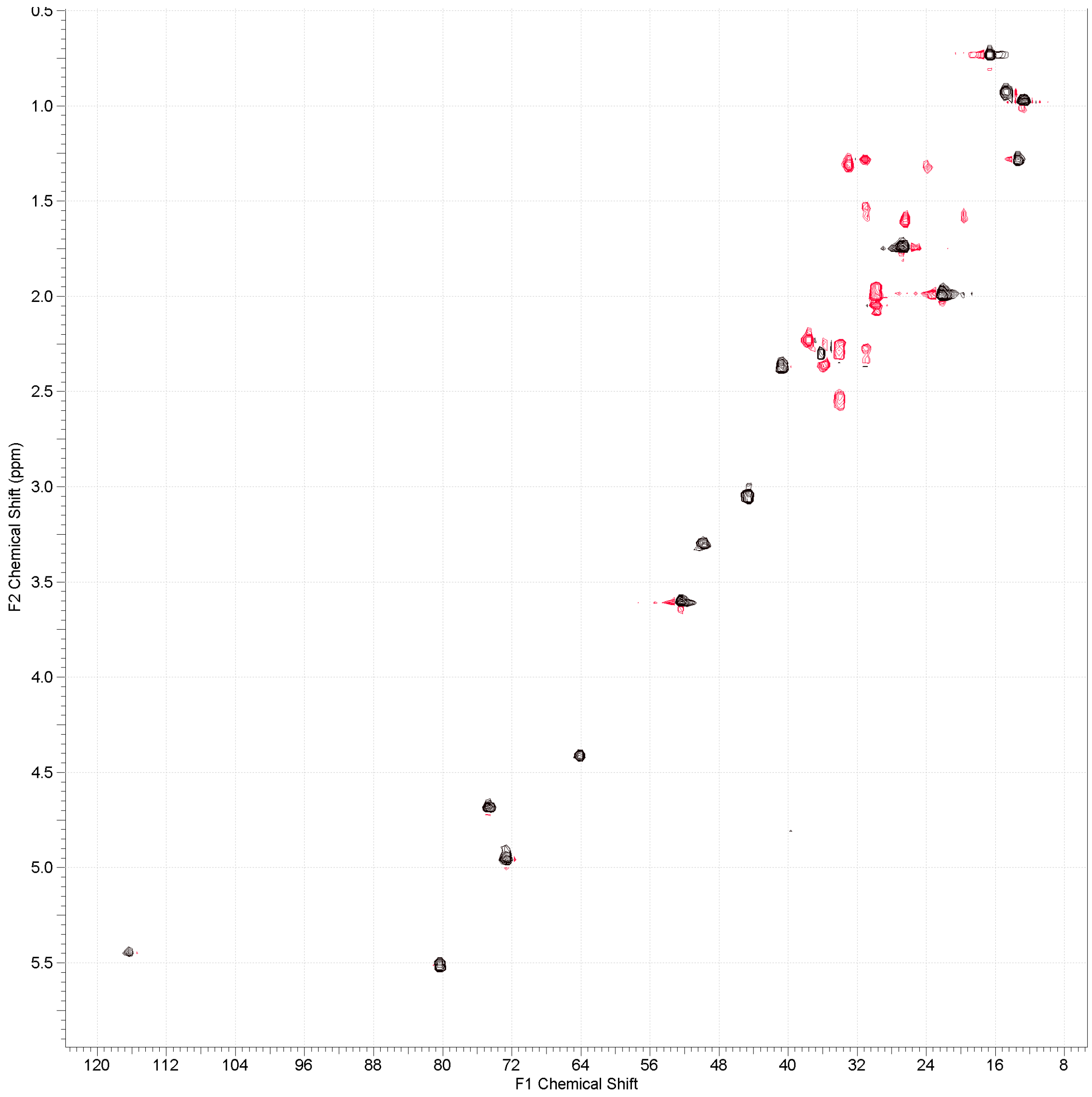

**Figure S10.** gHMBC NMR Spectrum of Briareolate Ester K (**2**) ($CD_3OD$, 400 MHz).

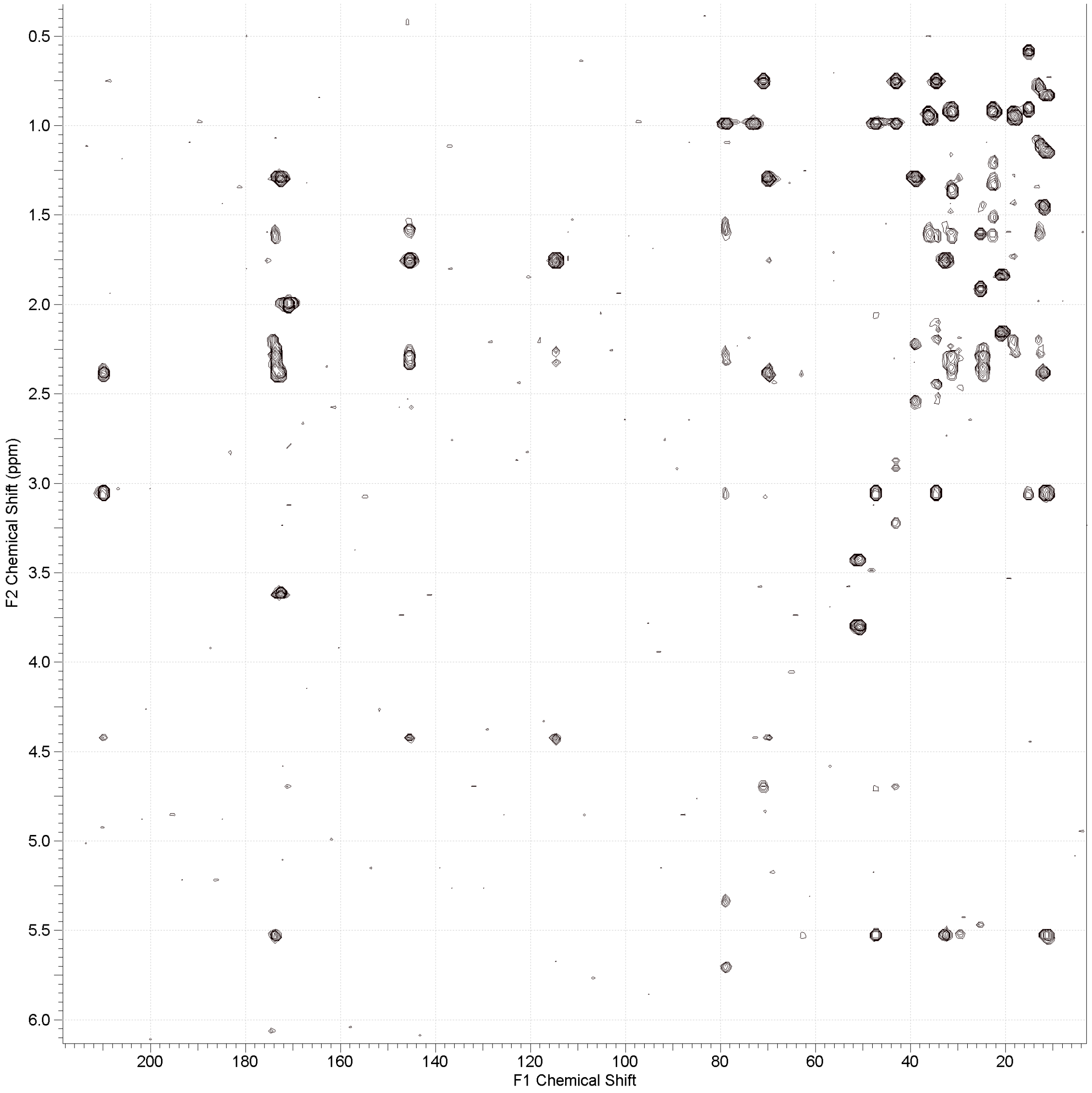

Supplement: Supplementary File 1: — PDF-Document (PDF, 681 KB) [file marinedrugs-10-01662-s001.pdf]
